# Occipital locked headache

**DOI:** 10.1186/1129-2377-14-S1-P181

**Published:** 2013-02-21

**Authors:** A Whitehouse, A Momoh-Ojewuyi, R Abusamara, S Al-Ani, R Alkilani, MAS Ahmed

**Affiliations:** 1 Barking Havering & Redbridge NHS Trust, UK

## Methods

Clinical data for 1029 patients (588 females; 627 Caucasians; mean age = 11.4 years) with headaches were prospectively examined in this hospital based study. Headache diagnosis was made on the basis of ICHD – II, 2004. 1. Headache diagnosis included migraine (n=598); tension type headaches (n=158); other headache types (n=91) and remained unclassified (n=182) patients. We have adopted previous descriptions of terms for anatomical sites for headache location. 2. Occipital locked headache (OLH) is defined as headache that is for all time fixed to the occipital region and never changed side.

## Results

48/1029 (4.7%) of patients experienced recurrent OLH during a mean headache course of 2.3 years. It was more for OLH to localise bilaterally (87.5%) as only four (8%) and two (4%) patients had right and left OLH respectively. Headache diagnosis was migraine (n=29); tension type headaches (n=5); and other headache types (n=5). Headache remained unclassified in 10/48 patients. Brain imaging was either normal (n=46) or showed no significant abnormalities (n=2).

## Discussion

In this study, sinister aetiologies of OLH were excluded among our patients. Primary headache was found to be the most common headache category among patients with OLH. Frequency of OLH was 5% and 4.4% patients with migraine and those with non-migraine headaches respectively.

## Conclusion

Primary headaches such as migraine and TTH are common causes of OLH, although OLH was infrequently found among patients with migraine and those with other primary headache types.
